# Plants and Light Manipulation: The Integrated Mineral System in Okra Leaves

**DOI:** 10.1002/advs.201600416

**Published:** 2017-02-26

**Authors:** Maria Pierantoni, Ron Tenne, Vlad Brumfeld, Vladimir Kiss, Dan Oron, Lia Addadi, Steve Weiner

**Affiliations:** ^1^ Department of Structural BiologyWeizmann Institute of ScienceRehovot76100Israel; ^2^ Department of Physics and Complex SystemsWeizmann Institute of ScienceRehovot76100Israel; ^3^ Department of Chemical Research SupportWeizmann Institute of ScienceRehovot76100Israel; ^4^ Department of Biomolecular SciencesWeizmann Institute of ScienceRehovot76100Israel

**Keywords:** calcium oxalate, microCT–confocal correlative microscopy, PDMPO, photosynthesis, silica

## Abstract

Calcium oxalate and silica minerals are common components of a variety of plant leaves. These minerals are found at different locations within the leaf, and there is little conclusive evidence about the functions they perform. Here tools are used from the fields of biology, optics, and imaging to investigate the distributions of calcium oxalate, silica minerals, and chloroplasts in okra leaves, in relation to their functions. A correlative approach is developed to simultaneously visualize calcium oxalates, silica minerals, chloroplasts, and leaf soft tissue in 3D without affecting the minerals or the organic components. This method shows that in okra leaves silica and calcium oxalates, together with chloroplasts, form a complex system with a highly regulated relative distribution. This distribution points to a significant role of oxalate and silica minerals to synergistically optimize the light regime in the leaf. The authors also show directly that the light scattered by the calcium oxalate crystals is utilized for photosynthesis, and that the ultraviolet component of light passing through silica bodies, is absorbed. This study thus demonstrates that calcium oxalates increase the illumination level into the underlying tissue by scattering the incoming light, and silica reduces the amount of UV radiation entering the tissue.

## 1 Introduction

Leaves are primarily organs for light utilization and carbon fixation.^1^, ^2^ Even though minerals (silica, calcium oxalates, and in some leaves also amorphous calcium carbonate) are often an integral part of different leaf tissues, they are not generally thought to be involved in optimizing the light absorption process, with documented exceptions for ficus and pecan leaves.^3^ Calcium oxalate (whewellite and weddellite) and to a lesser extent amorphous calcium carbonate (cystoliths) are widespread in a large variety of higher plant leaves.^4^ Many other roles, in addition to light manipulation, have been ascribed to calcium oxalates and cystoliths in leaves, including calcium regulation, leaf defense, detoxification of heavy metals, and an internal CO_2_ source.^5^, ^6^, ^7^, ^8^ Silica deposition (phytoliths) can occur in any part of the leaf epidermis and in the vascular tissue.^9^ Phytoliths are thought to play a role in many physiological processes such as reducing radiation damage, regulation of other nutrients, and pathogen resistance.^10^, ^11^, ^12^, ^13^, ^14^, ^15^, ^16^, ^17^, ^18^, ^19^ Silica is often considered to have a structural/defensive function.^20^, ^21^, ^22^ Mechanical properties of leaves are studied for leaves as whole units, with no special attention to the presence of silica or the other minerals.^23^, ^24^, ^25^

Minerals can be found throughout the leaf tissue and their locations are fundamental for understanding their function. The epidermis functions as a barrier between the outside and the leaf internal space. Epidermal cells are usually transparent allowing penetration of sunlight to the next tissue level, namely the palisade mesophyll tissue, where photosynthesis mainly occurs.^1^ The “spongy” mesophyll underlies the palisade mesophyll. Silica phytoliths are found in a variety of forms and locations in the epidermal tissue.^26^ Relatively large calcium oxalate and calcium carbonate bodies with very specific morphologies, sizes, and spatial distributions are often present in the palisade mesophyll^27^, ^28^ and in the spongy mesophyll along the veins.^29^, ^30^

The photosynthetic process occurs in chloroplasts, where the photosynthetic pigment chlorophyll captures the energy from sunlight and converts it into chemical energy. Because of the high absorption of chlorophyll in the top portion of the leaf, a light gradient is created from the leaf surface into the tissue inducing different light regimes. Under strong daylight illumination, the chloroplasts at the surface of the leaf are saturated, such that most of the light cannot be used for photosynthesis, but the chloroplasts in the depth of the leaf are light deprived.^31^, ^32^ In ficus plant leaves containing cystoliths, and in pecan leaves containing large calcium oxalate druses, the mineral deposits were proven to have a function in redistributing light into the leaf. Part of the light is scattered through the transparent mineral deposits in the bulk of the leaf, thus reaching the light deprived chloroplasts. This results in an increase in the yield of light utilization.^3^

In this study, we examine the optical functions of calcium oxalates and silica leaf minerals as an integrated system. Preserving the leaves as close to their natural state as possible, we use micro‐computed tomography (microCT) to image the leaf soft tissue and the distribution mainly of calcium oxalates, without fixing the tissue or using other invasive methods.^33^, ^34^, ^35^ MicroCT however cannot distinguish silica and soft tissue unless the silica phytoliths are large and relatively thick. It was therefore necessary to develop a different tool for examining the distribution of silica in the leaf. We report here a method to infiltrate the intact leaf with the {2(4pyridyl)‐5‐[(4‐(2‐dimethylaminoethylaminocarbamoyl)methoxy]phenyl}oxazole (PDMPO) fluorophore to study in situ the 3D distribution of silica deposits with confocal microscopy. PDMPO is a fluorophore that electrostatically interacts with the silica surface.^36^ PDMPO was used to investigate silica deposition in diatoms and radiolaria,^37^, ^38^, ^39^, ^40^, ^41^ and in plants to visualize extracted silica bodies.^42^ By superimposing the microCT and confocal images, we create a 3D map of the whole leaf showing the distributions of calcium oxalates, silica, and the naturally fluorescing chlorophyll.

Okra (*Abelmoschus esculentus*) leaves were chosen for the investigation of the functions and spatial interrelations of calcium oxalate druses and silica phytoliths. The large calcium oxalate monohydrate (whewellite) mineral aggregates, called druses,^43^ in the palisade mesophyll of okra leaves have the peculiarity of being four to five times larger than commonly observed druses.^4^ Small druses are deposited along the veins in the spongy mesophyll. Furthermore okra leaves have significant amounts of large silica phytoliths in both the upper and lower epidermis (LE). Thus the mineral types and spatial distributions in okra leaves are, for the most part, a good model for many other leaves. Here we use tools from the fields of biology, optics as well as light, electron, and X‐ray imaging in order to investigate the functions of the minerals in the leaves of okra.

## 2 Results

Our approach is to first study the overall spatial distribution of both calcium oxalate and silica minerals in large regions of leaves in order to better understand mineral function and aspects of control over mineral formation. To do this we developed a novel microCT–confocal microscopy correlative approach.

### 2.1 3D Visualization of the Minerals in Okra Leaves Using MicroCT

MicroCT is an excellent tool for visualizing the calcium oxalate minerals because of the large contrast difference between calcium containing minerals and plant tissue. The contrast between silica and plant tissue is weak, and as a result only the large silica bodies can be visualized.

**Figure 1:**
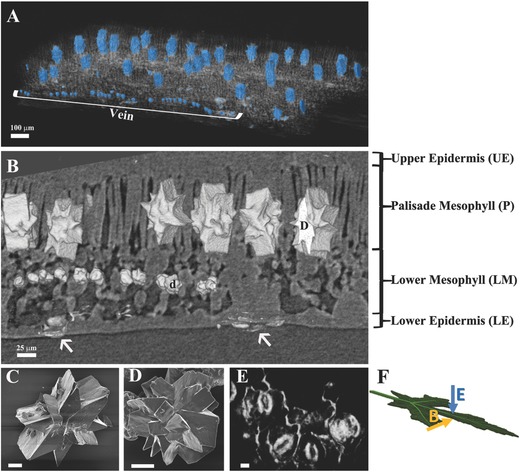
Minerals and soft tissue in the okra leaf. A) MicroCT 3D perspective view of calcium oxalate druses (colored in blue) in the leaf tissue. The vein is delineated by smaller druses. B) MicroCT reconstruction showing minerals in their physiological location. Druses (D) of about 100 µm are located in the palisade mesophyll (P). In the lower mesophyll (LM) smaller druses (*d*) of about 20 µm are located along a vein. In the lower epidermis (LE) it is possible to detect some silica phytoliths (white arrows). Note that in okra leaves phytoliths are very abundant even in the upper epidermis (UE). C) Scanning electron image of an extracted palisade druse. Palisade druses are star‐shaped bodies composed of eight to ten crystals, with high structural order. D) Scanning electron image of an extracted lower mesophyll druse. Mesophyll druses are less ordered spherical agglomerates of tens of crystals. E) MicroCT image of the phytoliths indicated by the right‐hand side arrow in (B), showing silica deposition in stomata and cell walls. C–E) scale bars 10 µm. F) Schematic representation of the direction in which the leaf was imaged in (B) (in cross‐section) and (E) (view from the lower epidermis side).

Okra leaves are characterized by two different types of druses (**Figure** **1**A,B). Large druses (about 100 µm × 50 µm × 20 µm) are distributed in the leaf palisade region at an average distance of 180 ± 60 µm (*n* = 125), corresponding to an average areal density of 50 druses cm^−2^. These large druses always grow as star‐shaped bodies, oriented perpendicular to the leaf surface with random orientation in the plane of the leaf. Okra palisade druses are each usually composed of between eight and ten crystals, which aggregate to form the star‐shaped body (Figure 1C). Rows of smaller druses (about 25 µm) are distributed in the lower spongy mesophyll along veins. The smaller druses have a less ordered spherical structure formed by tens of crystals (Figure 1D). Large silica phytoliths can be seen in the microCT acquisition (Figure 1E). These phytoliths consist of thick depositions of silica in cell walls and in stomata (pores found both in the upper and lower epidermis that allow movement of gases in and out of the intercellular spaces^44^).

### 2.2 Distribution of Silica and Chloroplasts Using Confocal Microscopy

The silica distribution in the leaf can be determined by staining silica with the green PDMPO fluorescent stain.

**Figure 2:**
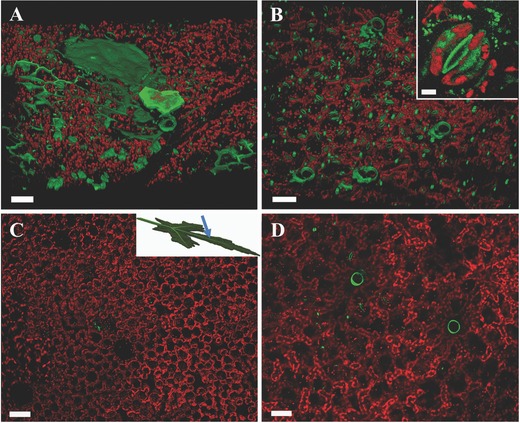
Confocal microscope images of the first few tens of µm of okra leaf portions stained with the PDMPO fluorophore. The leaf was imaged in a slightly tilted direction, shown in the inset in (C). Silica phytoliths marked with PDMPO are green while the chlorophyll autofluorescence is red. The volumes were slightly tilted from a top view to show the 3D of the silica structures. A,B) Areas of leaves from plants grown in silicic acid‐rich soil. C,D) Areas of leaves from plants grown in hydroponic conditions using a silicic acid free growth medium. The sections were chosen from the center of the leaves at the same stage of development. A) The upper epidermis leaf side. Silica deposition in the cell walls creates jigsaw puzzle structures. Occasionally, the silica bodies completely cover the upper part of several neighboring cells creating thin dome‐like structures. Smaller spherical phytoliths of about 5 µm are also visible. B) The lower epidermis leaf side. Silica deposition occurs in many stoma openings, in trichome (plant “hairs”) bases and in cell walls. Silica particles of about 5 µm are also visible. Inset: Silicification in the area of a stoma. The stoma aperture is completely silicified, while on the sides silica particles in the size range 0.5 ÷ 2.5 µm are deposited in the cell walls between chloroplasts. Scale bar 5 µm. C) The upper epidermis leaf side of hydroponically grown plants in the absence of silicic acid. Only traces of the dye are visible proving that PDMPO specifically stains silica and no other leaf component. D) The lower epidermis leaf side of the same leaf as in (C). Only very few trichome bases and stomata are visible. A–D) Scale bars 50 µm.

Pieces of fresh leaf were infiltrated with a solution containing PDMPO dye (**Figure** **2**A,B). The images show, in addition to stained silica, also the intrinsic red fluorescence of chlorophyll. The two channels were both excited at 402 nm.

A map of the chloroplast distribution in relation to the fluorescently labeled silica bodies was created. In the upper side of the leaf silica is deposited in cell walls in such a way as to produce large semicontinuous networks, with thin domes of silica covering several neighboring cells (Figure 2A). Small spherical depositions of 15–20 µm can also be observed. Silica is never covered by chloroplasts and is directly exposed to light. The tissue below the silica is rich in chlorophyll and consequently photosynthetically active. Note that in the imaged area a vein can be identified by the fact that no chloroplast is seen above it. Figure 2B shows smaller phytoliths, mostly hair bases and stomata, on the lower side of the leaf.

As PDMPO has not been used previously on intact leaves, we needed to verify that the dye binds only to silica. We therefore performed the same experiment on okra plants that were grown hydroponically in water with very little dissolved silicic acid (negative control). The negative controls showed practically no staining from the dye (Figure 2C,D) demonstrating that PDMPO does specifically stain silica and no other leaf component.

The use of PDMPO in intact fresh leaves also shows very small irregular silica particles. These small particles can be observed in cell walls, ether adjacent to one another, or isolated (inset in Figure 2). The small particles, which cannot be detected by microCT scans and are lost during phytolith extraction, are clearly a major component of the silica assemblage in okra leaves.

### 2.3 The Organization of Calcium Oxalates, Silica, and Chloroplasts: A MicroCT–Confocal Microscopy Correlative Approach

**Figure 3:**
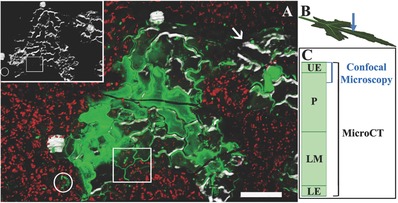
A) Overlay of microCT and confocal microscope images of the upper surface of a leaf showing the limitations of the two techniques. Inset: Same area shown only by microCT. Several upper epidermal neighboring cells are silicified. When silica deposition in the cell walls is still very thin (area highlighted in the rectangle) or when only spherical micron sized particles are deposited (area in the circle), phytoliths are visible only by confocal microscopy. The homogenous distribution of fluorescence within cells is attributed to silicification in upper cell walls visualized in the confocal microscope and not in microCT. The black line is due to a superficial crack in the epidermis. The microCT shows areas in which phytoliths are out of the confocal scan focus (arrow). The PDMPO is only slightly visible even if the silica deposition is thick. Scale bar 100 µm. B) Schematic representation of the direction in which the leaf was imaged, from the upper epidermis side. C) Scheme showing the depth of acquisition of microCT and confocal microscopy. UE = upper epidermis, P = palisade mesophyll, LM = lower mesophyll, LE = lower epidermis.

MicroCT and confocal microscopy provide complementary information about mineral location and structure, without requiring fixation or any preparation that significantly affects the tissue. The two techniques have certain limitations that can be partially overcome with a correlative approach. By looking at areas in which silica deposition is conspicuous enough to be seen in microCT volumes, it is possible to compare how silica is visualized by the two techniques (**Figure** **3**). The confocal technique is limited in the depth of the acquisition layer. When the sample is not completely flat (white arrow), some silica bodies that are visualized by microCT are not in the acquisition layer and are thus not imaged by confocal microscopy. In contrast, microCT provides information about the whole structure with no limitation due to the thickness or flatness of the sample, but the smaller phytoliths are not distinguishable. When the silica deposition in the cell walls is still very thin (area highlighted in the rectangle in the inset of Figure 3) or when only spherical micrometer‐sized particles are deposited (area in the circle in the inset of Figure 3), the structures are visible only in the confocal microscope (see the rectangle and circle in Figure 3).

**Figure 4:**
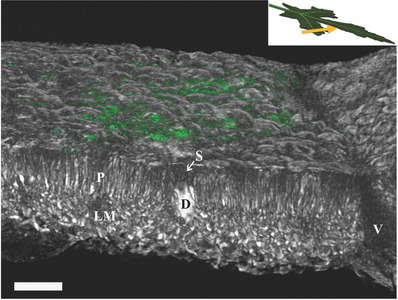
Overlay of microCT and confocal microscope volumes showing the surface and cross section of the leaf. The leaf was imaged in a slightly tilted direction, shown in the inset. A druse (D) extends from the palisade layer (P) cells to the lower mesophyll (LM) layer beneath. In the druse‐containing cell some free space (S) is always left between crystals and upper epidermis. In the lower mesophyll a big vein (V) runs perpendicular to the leaf section. Phytoliths (in green) are located in the upper epidermis just above the palisade layer. These thin silica deposits visible with the confocal microscope were not visible in the microCT scan of the same area. Scale bar 100 µm.

From the combination of microCT and confocal microscopy a 3D map that shows the mineral locations in the tissue can be obtained. **Figure** **4** is a microCT image of the surface and cross section of an okra leaf, showing the elongated palisade cells and the rounded cells in the underlying spongy mesophyll. A calcium oxalate druse (D) is visible at the interface between the palisade and spongy mesophyll layers. Note that the specialized druse‐containing cell contains some free space (S) between the crystals and the upper epidermis. In the lower mesophyll a large vein (V) runs perpendicular to the leaf section. By superimposing the confocal image onto the microCT image we can locate precisely the silica deposits (in green) in the upper epidermis just above the palisade layer. These silica deposits were not visible in the microCT image.

**Figure 5:**
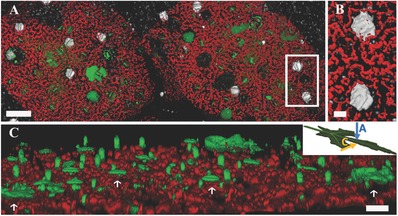
Relative distribution of oxalates, silica, and chloroplasts in the leaf. The directions in which the leaf was imaged in (A) (from the upper epidermis side) and in (C) (in cross section) are shown in the inset. A,B) Overlay of microCT and confocal microscope images showing the upper surface of a stained leaf. A) Reciprocal distribution of druses (white), phytoliths (green), and chloroplasts (red). Calcium oxalates almost never overlap with either silica or chloroplasts. Scale bar 100 µm. B) Magnification of the area in (A) defined in the rectangle. Chloroplasts are never located above druses and create a crown around the mineral even if a large empty space is left in the mesophyll above druses. Scale bar 10 µm. C) Confocal microscope 3D side view of a cross section of the upper surface of a leaf blade. Under the phytoliths (arrows) chloroplasts show the same distribution as in silica free tissue (see also Video S1, Supporting Information). Scale bar 25 µm.

**Figure** **5**A is a view of the calcium oxalate and silica distributions in the plane perpendicular to the upper leaf surface, namely in the direction from which the light reaches the leaf. This view shows the mutual distributions of druses, silica deposits, and chloroplasts. Significantly the calcium oxalates, the silica deposits, and the chloroplasts almost never overlap when viewed through the leaf surface (Figure 5A). A vein is located at the center of the image. Figure 5B shows a high magnification view of two druses and the surrounding chloroplasts. Chloroplasts are not located above druses even though there is always a large space above the druses which, in principle, could be filled by chloroplasts. Chloroplast distribution in the lower mesophyll under druses is uniform. Druses and phytoliths, in the most external layer of leaf tissue, are never covered by chloroplasts and they can be directly reached by light. Chloroplasts are regularly distributed under phytoliths in the palisade layer (Figure 5C and Video S1, Supporting Information).

We confirmed that silica and oxalates do not overlap using a colocalization test. This test examines the spatial anticorrelation between silica and oxalates by comparing the silica‐calcium oxalates relative distribution to possible random distributions created by scrambling the original images. In a scale from 0 to 1, where 0 is the random scenario and 1 is the case in which the two sets avoid each other. The value obtained for druses and phytoliths is 0.84 (**Table** **1**). This value is close to the perfect antilocalization distribution. The facts that silica and calcium oxalates do not overlap and that oxalate druses and silica deposits are not covered by photosynthetic tissue, are consistent with the hypothesis that one of the functions of these minerals is to interact with the incoming light.

**Table 1:** Results of the colocalization test. The statistical test compares the correlation coefficient (Robs) of the druses and silica. The scale ranges from 0 to 1, where 0 is a random distribution of silica and calcium oxalates and 1 is the case in which the two sets perfectly avoid each other

Region	Number of druses	Area [mm^2^]		*R*_obs_
1	7	1.3		0.86
2	11	1.1		0.91
3	5	1.2		0.84
4	12	2.1		0.97
5	7	1.4		0.96
6	10	3.6		0.74
7	6	1.6		0.62
Average	58	12.3		0.84

### 2.4 Optical Studies to Test the Hypothesis That One Function of Calcium Oxalates and Silica in Leaves Is to Interact with Incoming Light

The observations that silica bodies are never located above druses, and that the chlorophyll distribution is such that it does not block light entering the druses or the silica bodies, are both consistent with the hypothesis that one function of the minerals is to interact with the incoming light. To further test if druses in the okra leaf do enhance the photosynthetic efficiency of the leaf, as was previously demonstrated for cystoliths and druses in other leaves,^3^ we carried out an experiment in which chlorophyll fluorescence is measured as a proxy of the unused illumination and therefore of the light harvesting efficiency of the photosynthetic apparatus.

Fluorescence is the result of the reemission of photons that are not used by the photosynthetic machinery and is thus strongly enhanced by its saturation.^45^ Fluorescence emission is therefore a measure of the “wasted light”, and its intensity is inversely proportional to the photosynthetic efficiency. In leaves the palisade cells usually absorb most of the light reaching the leaf and screen the lower mesophyll from illumination.^46^ A strong light gradient is created inside the tissue and the steep gradient limits the photosynthetic efficiency of the whole mesophyll under saturation.^32^ If the druses scatter light in the leaf, making it more available to otherwise screened chloroplasts, the total emitted fluorescence should decrease.

**Figure 6:**
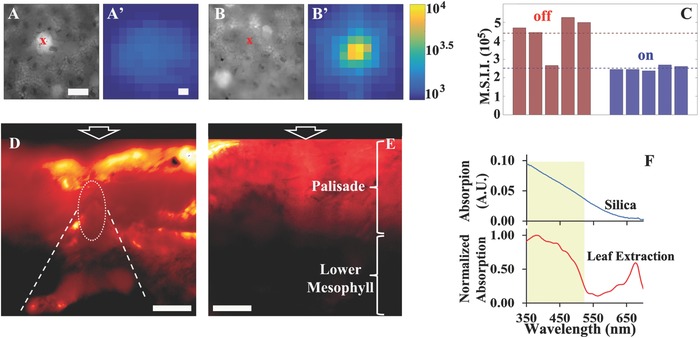
Optical function of druses and silica. A–C) Microfluorimetry measurements on leaf portions. A,B) Transmitted wide field light images of the locations chosen for measurement (*x*). The white spot in (A) is a druse. Scale bar 100 µm. A′,B′) Logarithmic color scale representation of measured chlorophyll autofluorescence intensity after laser excitation. Color reference scale is on the right side. The laser diameter was 15 µm while the fluorescence was detected from an area of 0.03 mm^2^. Scale bar 20 µm. A′) Measurement “on druse” and B′) measurement “off druse”. C) Bar plot of the maximal spatially integrated intensity (M.S.I.I.) obtained from microfluorimetry curves of on druses versus off druses locations. D,E) Detection of the light distribution in the leaf tissue, seen in cross‐section. Images showing an intensity color map of the fluorescence induced by a red laser. Color scale: increasing intensity from black to yellow. The fluorescence intensities in (D) and (E) are in the same color scale. The arrows indicate the incoming light direction. D) When a druse (dashed circle) is present part of the light is channeled into the lower mesophyll producing a cone of light. Scale bar 50 µm. E) Fluorescence is strong only in the palisade. Most of the photosynthesis takes place within this layer. Scale bar 50 µm. F) Absorption spectra of extracted silica (blue line) and of extracted chlorophyll and pigments (red line). Silica bodies absorb light mainly in the UV region, but absorption is already substantial below 530 nm, overlapping with the absorption peak of chlorophyll and pigments (yellow rectangle). The absorption of silica was measured for one layer of phytoliths.

We performed a microfluorimetry experiment by using a focused laser beam (15 µm diameter) to directly illuminate druses (“on druse”) located through transmittance images as well as control areas that did not contain druses (“off druse”) (**Figure** **6**A,B). Chlorophyll fluorescence is then imaged onto a charged coupled device camera. While in “on druse” locations (Figure 6A′) the emission is dim and arises from an area larger than the illumination spot, “off druse” (Figure 6B′) locations present a maximum intensity higher by a factor of ≈100 and a fluorescence spot comparable in size to that of the laser beam. By integrating the images we obtain the total intensity of fluorescence signal collected through our objective lens. Figure 6C presents four representative integrated intensity measurements for “on druse” and “off druse” locations at the time of maximal fluorescence. The “off druse” locations show a factor of two to three higher integrated fluorescence intensities in comparison to the “on druse” locations. This marked effect provides supporting evidence that druses scatter the incoming light and therefore mitigate the saturation of the chloroplasts, thus reducing the amount of photons not used in photosynthesis. A mistaken identification of “off druse” locations that missed the presence of druses during preliminary transmission observations may explain the rare occurrence of low intensity measurements in the “off druse” measurements.

In order to rule out geometrical differences at the “off druse” and “on druse” locations as the sole reason for the large fluorescence intensity differences, we calculated the light gradient and fluorescence collection efficiency expected by an extended version of the Munk–Kulbeka model^47^ (see the Supporting Information for complete details). The original model calculates the intensity profile of the exciting light within the leaf as a result of scattering and absorption within the leaf tissue. Here, we extend the model to obtain the fluorescence light. The model shows that most of the light entering the leaf, 98% and 85% for “off” and “on” druse positions respectively, is absorbed by chlorophyll (Figure S3, Supporting Information). To model the light intensity that escapes the upper epidermis layer into the objective lens, we calculate the backward flux of fluorescence light in this layer for both “off” and “on” druse positions (Figure S4, Supporting Information). Calculated values differ by ≈5%, showing that the substantial effect that we observe (100% difference) cannot stem only from the different thicknesses of mesophyll at the two locations. Intuitively, since the leaf is approximately three absorption lengths thick, most of the contribution to fluorescence is from the topmost parts of the leaf, roughly a single absorption length deep. Thus, the extra thickness at “off druse” locations does not significantly affect the fluorescence signal.

In order to directly visualize the druse scattering function we built a setup which allows imaging the chlorophyll fluorescence distribution in different tissues inside the leaf. We adapted the procedure used in previous studies.^48^, ^49^, ^50^ Leaves are cut perpendicular to the leaf blade to expose a cross section of the whole leaf width. Laser light passes through a slit and impinges on the upper leaf blade natural surface very close to the cut. We studied the case in which light is shone on a druse and compared the result with what was obtained from areas devoid of minerals.

Figure 6D,E shows an intensity color map of the fluorescence induced in the tissue when the red laser hits the leaf blade. Chlorophyll fluorescence is strong in the palisade indicating that most of the photosynthesis is taking place within this layer. Druses were located using the epifluorescence microscope binocular. When a druse is present (Figure 6D) part of the light is channeled into the lower mesophyll. The produced cone of light indicates that chlorophyll activity is taking place even below the palisade layer. When an area without druses is imaged fluorescence can be detected almost exclusively in the palisade layer and not in the lower mesophyll (Figure 6E). We therefore conclude that calcium oxalate druses function as scatterers, channeling the light to the lower mesophyll and by so doing increase the photosynthetically active volume of the leaf. The regular organization in the tissue and the tilted orientations of the druses seem to be tailored to best perform the function of evenly redistributing light into the lower mesophyll. In fact the average distance between nearest‐neighbor druses is 180 ± 60 µm and the measured diameter for the scattering cones is 116 ± 12 µm (*n* = 10). This means that druse distribution creates a system in which a large fraction of the lower mesophyll is reached by scattered light maximizing the amount of photosynthetic active tissue.

Confocal imaging showed that the silica content in okra leaves is higher than expected when based only on the large silica phytolith distribution observed by microCT. There are many micrometer size silica bodies distributed throughout the epidermis covering wide areas of the leaves. Silica deposits can absorb light, and especially small spherical particles can scatter back excess radiation. We measured the leaf silica absorbance after extracting intact phytoliths and depositing them as a single layer on a quartz slide. Silica mainly absorbs and scatters in the UV region, but the absorption is already substantial at 530 nm and below, thus partially overlapping with the main absorption peak of chlorophyll and leaf pigments (Figure 6F). By taking into account the silica absorption and scattering characteristics and the observed distribution of silica in relation to chlorophyll and large druses, we propose that one function of large and small silica bodies is to protect the underlying biological tissues from UV damage. Furthermore, the silica bodies do not overlap with the large druses, such that the amount of light scattered by the druses and used for photosynthesis in the lower mesophyll is not reduced.

## 3 Discussion and Conclusions

A detailed 3D map of the mineral distribution in the okra leaf soft tissue was obtained using the novel correlative microCT–confocal microscopy method. Silica phytoliths and the large calcium oxalate druses, as well as the chloroplasts, form a complex system with a well‐defined relative distribution. This distribution points to a significant role of the minerals in light manipulation. This does not exclude the possibility that the minerals also perform other functions. Direct visualization shows that in okra leaves palisade druses function as light scatterers channeling light into the lower mesophyll. Microfluorimetry together with the light directionality simulation show that this scattered light is utilized for photosynthesis and the unused portion of incident light is reduced. Based on the measured average distance between large druses as well as the diameters of the light cone produced by scattering, we conclude that in okra leaves almost all the lower mesophyll is illuminated by scattered light. As a result the steep light gradient is reduced and the photosynthetic efficiency is increased.

The scattering function of cystoliths and other oxalate druses was already demonstrated for leaves of pecan trees.^3^ Here we show that calcium oxalate druses can function as light scatterers even in the case of an annual herbaceous plant. Together all these observations point to the fact that calcium oxalates and cystoliths functioning as light manipulators could be a widespread phenomenon.

In this study we focus on the palisade druses. Note however that small druses in okra leaves are located along the veins, which are never covered by chloroplasts. It would therefore be interesting to determine whether or not the small druses also scatter light for photosynthesis. However, the small druses might also function in protecting the vein content by scattering incoming light, in mechanical reinforcement, in defense against insects, or all of the above.

The large silica bodies (phytoliths) in the epithelial layer, most probably provide protection from UV radiation to the palisade cells. Significant amounts of silica are in the form of small particles. The presence of the silica is important not only to avoid tissue damage but also to prevent photoinhibition which results in the reduction of the photosynthetic capacity of the leaf.^51^ In fact significant photoinhibition was proven to occur even at relatively low light intensity when leaves are exposed to UV, forcing plants to constantly repair the damage.^52^ We have observed that silica deposits are detectable even in newly formed leaves (unpublished observations). This would agree with a function of silica in protecting the photosynthetic soft tissue from the very first stages of the leaf life.

Together with UV, a small amount of visible light that could be used for photosynthesis is absorbed and scattered by biogenic silica. This would probably not be important for photosynthesis in the palisade layer, which is already saturated under normal light conditions. However, based on the fact that silica does not cover the large druses, this portion of light could be important for harvesting photons in the mesophyll layer.

The microCT–confocal microscopy correlative method is a novel technique that can be used to image minerals in soft tissues in 3D, including not only calcium oxalates and silica but also amorphous calcium carbonate cystoliths. This method does not require fixation or other invasive preparations of the sample and it allows imaging leaf components without significantly altering the natural conditions. This approach provides a new view of the regulated event of mineralization. The correlative method that we propose can be applied for other studies on function and formation of biominerals and to investigate different leaf species and plant tissues.

In conclusion, the correlative microCT and fluorescent confocal images show that the distributions of calcium oxalate druses, silica deposits, and chlorophyll are consistent with the minerals fulfilling functions in manipulating light. We show that the light scattered by the calcium oxalate druses is used for photosynthesis, whereas the silica deposits may protect the plant tissue from UV radiation.

## 4 Experimental Section

*Plant Material*: Fresh leaves were collected from okra (*Abelmoschus esculentus*) plants grown in the Weizmann Institute greenhouses. Silica free plants were grown in hydroponic conditions. The system consisted of a dark sealed box. Holes to allow the plant growth were drilled in the box cover. Plastic foam was placed around the stems to reduce the evaporation of the nutrient solution without constraining the plants. The silicon free nutrient solution contained 2.5 × 10^−3^ m KNO_3_, 1.5 × 10^−3^ m Ca(NO_3_)_2_, 0.1 × 10^−3^ m NH_4_H_2_PO_4_, 0.8 × 10^−3^ m MgSO_4_, 0.18 × 10^−3^ m CuSO_4_, 4.5 × 10^−6^ m Na_2_MoO_4_, 0.5 × 10^−6^ m ZnSO_4_, 4.5 × 10^−6^ m MnCl_2_, 22 × 10^−6^ m H_3_BO_3_, and 10 × 10^−6^ m EDFS (C_10_H_12_N_2_NaFeO_8_). The solution was constantly aerated using an aquarium pump.

*Mineral Extraction*: Method 1: Calcium oxalates were extracted from the leaves following Arnott et al.^43^ Leaves were carefully washed, then cut into small pieces and blended in absolute ethanol for 2 min. The product was filtered through a gauze filter. The filtrate was centrifuged in order to remove the supernatant. The pellet was resuspended and vortexed in ethanol. This was repeated twice with ethanol and then again three times with deionized water (DW). The pellet was mixed with sodium polytungstate (3Na_2_WO_4_′9WO_3_′H_2_O) of 1.7 g mL^−1^ density and centrifuged three times. The supernatant, together with organic material that does not pellet under these conditions, was discarded and the pellet was resuspended, vortexed, and centrifuged again three times with DW, then with absolute ethanol.

Method 2: Leaves were cut in pieces and placed in a ceramic mortar. The mortar was heated in a Lindberg Bluem laboratory oven which was set at 500 °C for 5 h. Then the ashes were dissolved in 1 n HCl and washed in DW and then acetone.

*Leaf extracts* containing chlorophyll and pigments were obtained by blending leaves and filtering the organic through a gauze filter as for mineral extraction. The filtrated solution was then centrifuged. In this case, the supernatant was kept and the precipitate was eliminated in order to remove the small particles that could not be filtered.

*Scanning Electron Microscopy*: The extracted minerals were deposited on a microscope stub and carbon coated. Images were obtained using a SUPRA55 scanning electron microscope (Zeiss).

*MicroCT*: MicroCT scans were acquired using a Micro XCT‐400 (Zeiss X‐ray Microscopy, California, USA). Triangular sections of leaves (base 1 cm, length 2 cm) were cut and placed individually in a plastic pipette tip, which was previously sealed by melting the extremity using a flame. To prevent dehydration, the tips were filled with DW leaving part of the leaf in air and the tip in water. Only the part of the leaf not immersed in water was imaged. This increases the contrast and improves the final resolution without drying the leaf tissue. The tomographic image was obtained by taking 1500 projections over 1800 at 40 KV and 200 µA. The final pixel size was 2 µm.

*Confocal Microscopy*: In order to image phytoliths in confocal microscopy we used 0.125 × 10^−6^ m PDMPO (a fluorescent silica marker, LysoSensor Yellow/Blue DND‐160, ThermoFisher Scientific) to label the silica.^39^, ^42^, ^53^, ^54^ We confirmed that PDMPO does indeed stain extracted phytoliths by using confocal microscopy (Figure S1, Supporting Information).

We modified the previously reported methods^42^, ^54^ to infiltrate entire leaf sections (1 cm × 2.5 cm) in order to stain silica in the leaves without extraction. Leaf sections were immersed in a petri dish containing the dye solution. The solution was infiltrated into the leaf by placing the petri dish in a desiccator connected to a vacuum pump for 30 min, whereupon the air is removed from the leaf tissue and the solution is taken up through the leaf veins. The infiltrated leaf portions were carefully washed in tap water, placed between coverslips that were sealed with silicon grease and nail polish to keep the leaf hydrated. The marked samples were imaged on both sides with a confocal FW1000 laser scanning microscope, Olympus IX81. The excitation laser wavelength was 402 nm for both the chlorophyll and the PDMPO dye. The emission was taken with a 520–550 nm band‐pass filter in the case of the PDMPO, whereas the band‐pass filter was 655–755 nm for the chlorophyll. The volumes were acquired with 4×, 10×, 20×, and 60× oil objective lens magnification, with *z* steps of 25, 4, 1.5, and 0.8 µm, respectively. Note that at 20× and 60× magnifications only the first tens of micrometers of leaf can be imaged.

We tested that the recorded green emission was due to the PDMPO dye and not the sample autofluorescence by changing excitation wavelength and by measuring the emission peak on phytoliths, on chloroplasts and in background areas (Figure S2, Supporting Information).

*Confocal Microscopy–MicroCT Correlative Approach*: Rectangular sections were cut from leaves at different stages of development. A distinctive area of a hundred square micrometers was marked using silver conducting adhesive from Electron Microscopy Sciences. The sections were placed in a polypropylene rectangular custom made container (internal size: 1.7 mm × 7.7 mm × 29.7 mm, thickness: 2 mm) connected to the microCT stage which keeps the sample straight and vertical. One of the leaf section extremities was kept in water while the scan was performed above, in air. After the microCT scan the samples were imaged on both sides in the confocal microscope as previously described.

The two sets of data were then superimposed manually using the silver adhesive and veins as fiducial points which are visible both in the microCT and in the confocal microscope images. 3D volumes were produced using the Avizo 3D analysis software.

*Colocalization Test*: In order to perform a colocalization test, calcium oxalates and silica were imaged perpendicular to the upper leaf blade surface using two different channels. To perform the test we used the colocalization Test plugin of ImageJ. The statistical test compares the correlation coefficient (*R*_obs_) of the two channels with one of the channels spatially shifted (*R*_rand_).^55^ Whole original images were analyzed. The randomization method used was Costes approximation. We used a manual point spread function of 10 pixels and 50 iterations. The intensity of colocalization was expressed by the Pearson's correlation coefficient which is not sensitive to differences in signal intensities.^56^ We considered only the random and anticorrelation cases. Scale: 0 = no correlation, 1 = perfect anticorrelation.

*Scattering Measurements*: Leaf sections were cut perpendicular to the leaf surface and mounted on a custom made stage. The setup was modified from Takahashi et al.^50^ The imaging system was composed of a Delta Vision Deconvolution epifluorescence microscope equipped with an additional diode laser (660 nm, 80 mW) mounted perpendicular to the detector (Olympus IX71 Microscope). The laser light passed through a slit and impinged on the upper leaf surface. The stage was designed to keep the section in the right geometry so that the upper leaf surface faces the laser beam and the cut tissue faces the microscope objective. Chlorophyll fluorescence generated by the laser in the leaf tissue was imaged with a 10× objective using a Cy5 filter.

*Modulated Fluorimetry*: To perform leaf fluorescence measurements a microfluorimeter setup was custom built around a commercial microscope (Eclipse Ti‐U, Nikon). A 635 nm pulsed diode laser (EPL635, Edinburgh Instruments) emitting 100 ps short pulses at a 2 MHz repetition rate excited the Chlorophyll b molecules in the leaf. The beam was focused through an objective lens (20X, 0.4 N.A., Nikon) onto a ≈15 µm diameter spot. Control over the laser excitation power was achieved by using a variable retarder between two linear polarizers and a mechanical shutter was used to start and stop it. Fluorescence light was collected through the same objective, filtered from the illumination by a dichroic mirror (650LP Semrock) and a dielectric filter (635LP Semrock), and imaged onto an electron‐multiplying charge‐coupled device camera (iXon Ultra 897, Andor). The camera captured a ≈40 s long frame series with a ≈20 ms exposure time to accurately capture the fluorescence dynamics. Dynamic traces started with a dim excitation (typically 0.1 nW) with a 5 s duration in order to obtain the photoluminescence (PL) quantum yield for unsaturated reaction centers followed by a 20–40 s dark followed by another period of saturating excitation power (typically 5 nW). The data were recorded with the Solis software and analyzed with a custom built MATLAB script.

*Spectrophotometry*: The absorption spectra were collected with an Olis Clarity 1000 UV/VIS spectrophotometer, or with a Quantaurus QY model C11347‐11 manufactured by Hamamatsu. The measurements with the two techniques yielded the same results. Phytoliths were extracted as described in the second method for mineral extraction. The solution containing phytoliths was then deposited on a quartz slide which was cut to enter the spectrophotometer chamber. During the deposition the slide was heated on a hot plate, so that the solvent could evaporate immediately. This precaution prevented the phytoliths from aggregating together during a slow solvent evaporation. The leaf extract was diluted in acetone, dropped on a quartz slide as for phytoliths, and dried.

## Supporting information

As a service to our authors and readers, this journal provides supporting information supplied by the authors. Such materials are peer reviewed and may be re‐organized for online delivery, but are not copy‐edited or typeset. Technical support issues arising from supporting information (other than missing files) should be addressed to the authors.

Supplementary

Supplementary
