# Supplementary material for: Plants and Light Manipulation: The Integrated Mineral System in Okra Leaves
Source: Adv Sci (Weinh). 2017 Feb 26;4(5):1600416. doi: 10.1002/advs.201600416 (PMC5441490; doi:10.1002/advs.201600416)
Supplement: Supplementary file 1 — Supplementary [file ADVS-4-na-s001.pdf]

Copyright WILEY-VCH Verlag GmbH & Co. KGaA, 69469 Weinheim, Germany, 2017.

ADVANCED SCIENCE
Open Access

Supporting Information

for *Adv. Sci.,* DOI: 10.1002/advs.201600416

Plants and Light Manipulation: The Integrated Mineral System in Okra Leaves

Maria Pierantoni, Ron Tenne, Vlad Brumfeld, Vladimir Kiss, Dan Oron, Lia Addadi, and Steve Weiner**

Supporting Information

Plants and Light Manipulation: the Integrative Mineral System in Okra Leaves

Maria Pierantoni, Ron Tenne, Vlad Brumfeld, Vladimir Kiss, Dan Oron, Lia Addadi and Steve Weiner**

Video S1. Superimposed microCT and confocal microscope images. MicroCT components are in white, confocal microscope components are in green for the silica stained with PDMPO and red for the chlorophyll autofluorescence. The video shows the view of the leaf surface with all 3 components. The image stack is then rotated 90°.

Pause 1: Side view of the leaf. Note that the components imaged by the confocal microscope are limited to the upper 40 microns. Note that chlorophyll underlies the silica mineral distribution. Note too the locations of the palisade druses and the small druses lining the vein.

The image is then rotated another 90 degrees and then the silica is removed digitally layer by layer from the upper surface to reveal the underlying chloroplasts.

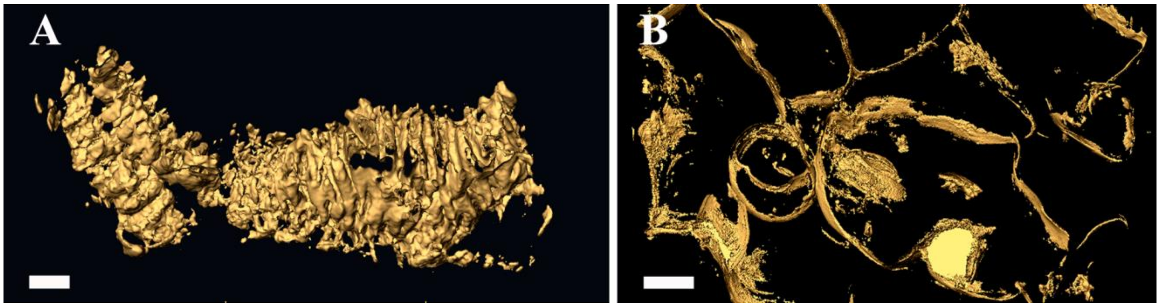

Figure S1. Extracted phytoliths. The phytoliths were stained with PDMPO solution and 3D imaged by confocal microscopy. (A) phytolith extracted form a vein, (B) silicified cell walls. Scale bars: (A) 20µm, (B) 10µm.

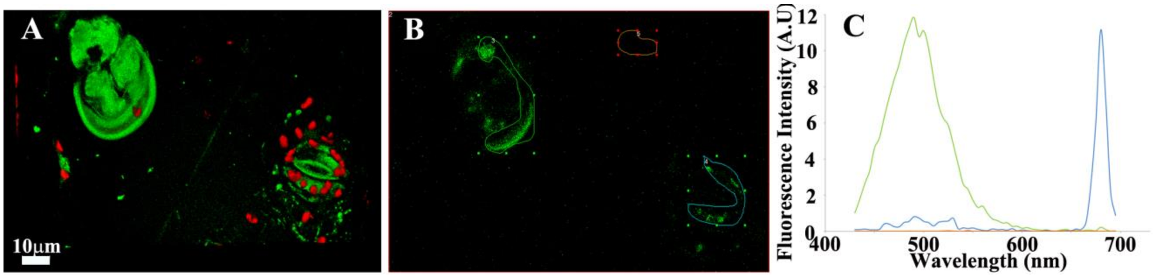

Figure S2. Testing the emission wavelength for different locations. (A) Confocal image (B) the selections show where the emission spectra were acquired, (C) Emission spectra of the three selected area obtained exciting at 402 nm. The emission was measured for a phytoliths without visible chlorophyll around (green selection), for a stoma and several chloroplasts (blue selection) and for an area were no signal is visible (red selection) The PDMPO is mostly responsible for the 500 nm peak of emission, chlorophyll contributes only weekly to the 500nm peak.

Munk-Kulbeka model for leaf PL measurements

The model, explained in detail below, follows closely the work done by Simonot *et. Al* [1].

In this one dimensional model light goes either in the forward or backward direction. Light can undergo backward scattering and switch its direction by 180 degrees (S) or absorption (K). Forward scattering is considered here as a portion of the transmitance. The profile of the excitation light is therefore calculated by the solving of the following set of ordinary differential equations:

(Eq. 1a) $$\frac{dI_A}{dx} = -(S + K)I_A + S \cdot J_A$$

(Eq. 1b) $$\frac{dJ_A}{dx} = (S + K)J_A - S \cdot I_A$$

Where, x is the direction perpendicular to the leaf surface. $x = 0$ is the upper epidermis layer and $x = 1$ is the lower epidermis layer. $I_A(J_A)$ is the forward (backward) advancing excitation light power flux. S and K are the scattering and absorption coefficients per one leaf thickness respectively taken from the experimental work done by Gausman and Allen [2] as 0.6 and 2.6 respectively.

The absorption coefficient is corrected to account for the presence of the crystals in the leaf. The experimental value measured by Gausman and Allen is an average over the whole leaf area including weakly-absorptive sections covered by the druses. In the results below the area fraction covered by drusesis taken as 0.05, yielding an absorption coefficient of 3.25.

The boundary conditions for the solution of Eq. 1a,b are:

(Eq. 2)
$$I_A(0) = 1$$
$$J_A(1) = R_0 \cdot I_A(1)$$

Where R_0, the reflection of the bottom layer of the leaf is taken as 0.04. The intensity of the impinging light is normalized to 1. For the “on druse” position the second boundary condition is given for $x = \frac{1}{2}$ instead of $x = 1$.

The solutions for both $I_A$and J_A are a sum of a rising and decaying exponentials and are presented in Figure S3 below for the “on druse” and ”off druse” positions:

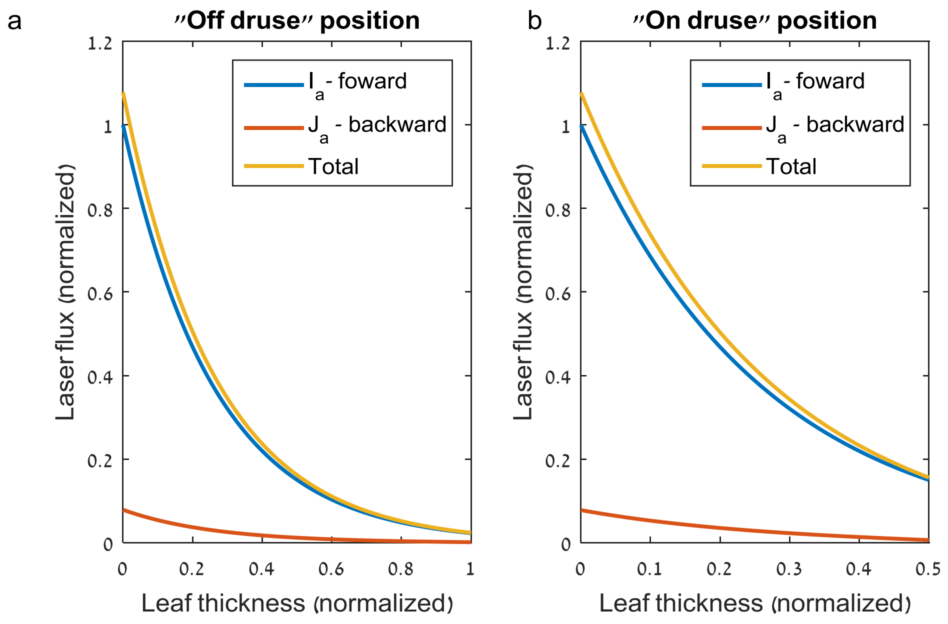

Figure S3. Excitation light profiles. Excitation light profiles are calculated from solutions to the K-M model as presented in Eq. 1-2. (A) Solution for the case of a "off druse" position (B) Solution of the "on druse" position. A thickness of 0.5 leaf thicknesses is taken as the boundary for the "on druse" position.

Since the absorption is quite strong in both cases more than 90% of the absorbed light is localized in the top half of the leaf. 15% of the incoming light is transmitted through the "on druse" position while only ~2% through the "off druse" position. In both cases ~8% of the light is backscattered.

Using the solution for the excitation profile as the source for fluorescence within the leaf one can write equations for the fluorescence light intensities in the forward and backward directions:

(Eq. 3a) $$\frac{dI_E}{dx} = -(S_E + K_E)I_E + S_E \cdot J_E + 0.5 \cdot QY \cdot K(I_A + J_A) + 0.5 \cdot QY \cdot K_E(I_E + J_E)$$

(Eq. 3b) $$\frac{dJ_E}{dx} = (S_E + K_E)J_E - S_E \cdot I_E - 0.5 \cdot QY \cdot K(I_A + J_A) - 0.5 \cdot QY \cdot K(I_E + J_E)$$

Parameters carrying a subscript E characterize the emission wavelengths following the same definitions given above. Although the model can account for parameters of different magnitude for the absorption and emission wavelengths we chose to use equal values due to the proximity of the laser and PL wavelengths, i.e. $K_E = K, S_E = S$.

The fluorescence term here assumes a steady state in the population of excited chlorophyll (i.e. a small change on the time scale in which light travels through the leaf). The quantum yield (QY) of the chlorophyll under saturation is taken as 10%.

The boundary conditions for these equations are similar to the ones given in Eq. 2 with the exception that in this case there should be no downward flux of fluorescence at the upper epidermal layer:

(Eq. 4) $$I_A(0) = 0$$
$$J_A(1) = R_0 \cdot I_A(1)$$

Again, for the 'on' crystal position the second boundary condition is given for $x = \frac{1}{2}$ instead of $x = 1$.

Figure S4 shows the profile of luminescence light within the leaf for "on druse" and "off druse" positions divided into forward and backward intensities.

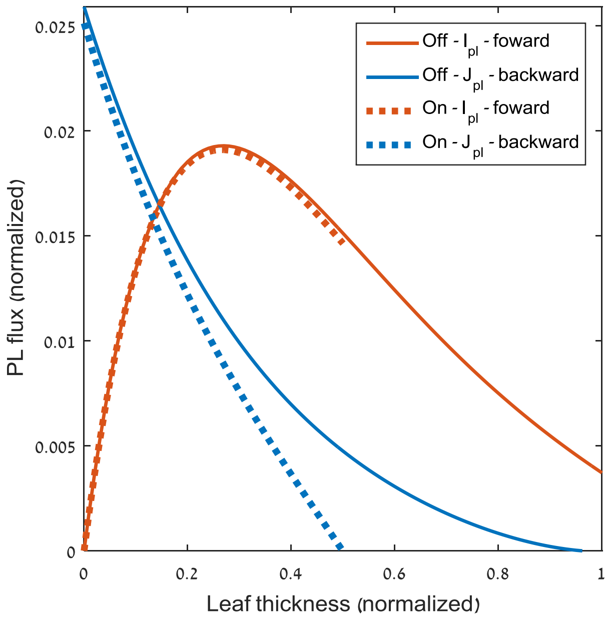

Figure S4. Fluorescence light flux distribution within the thickness of the leaf. The light flux is calculated for the "off druse" (solid lines) and "on druse" (dashed lines) positions. The forwards and backwards directed fluxes are shown in blue and red lines respectively. The backwards fluorescence flux at $x = 0$ is proportional to the signal measured in our setup.

Here we are mainly interested in the value of J_E at $x = 0$ which is the PL photon flux directed towards our collection lens. The values in both positions differ by less than 5% supporting the claim that the difference in the PL intensity in our measurements is not due to different effective leaf chlorophyll thicknesses in the two positions.

We note that these values model the measured PL intensities already taking into account the excitation light profile. Meaning, it already considers the fact that a bigger fraction of excitation light is transmitted in the "on druse" position.

References

1. Simonot L, Thoury M, Delaney J Extension of the Kubelka-Munk theory for fluorescent turbid media to a nonopaque layer on a background. J Opt Soc Am A 2011 28: 1349-1357.

2. Gausman HW, Allen WA Optical parameters of leaves of 30 plant species. Plant Physiol 1973 52: 57-62.
